# Hepatocellular carcinoma surveillance, incidence, and tumor doubling times in patients cured of hepatitis C

**DOI:** 10.1002/cam4.4508

**Published:** 2022-03-09

**Authors:** Ponni V. Perumalswami, Brooke Wyatt, Chip A. Bowman, Krupa Patel, Anna Mageras, Sara C. Lewis, Andrea D. Branch

**Affiliations:** ^1^ Division of Liver Diseases Icahn School of Medicine at Mount Sinai New York New York USA; ^2^ Division of Hospital Medicine Icahn School of Medicine at Mount Sinai New York New York USA; ^3^ Department of Radiology Icahn School of Medicine at Mount Sinai New York New York USA

**Keywords:** African American, direct acting antiviral, hepatitis C, hepatocellular carcinoma, LI‐RADS, sustained virological response

## Abstract

**Background:**

Hepatocellular carcinoma (HCC) incidence and mortality vary by race/ethnicity and both are higher in Black patients than in Whites. For HCC surveillance, all cirrhotic patients are advised to undergo lifelong twice‐annual abdominal imaging. We investigated factors associated with surveillance and HCC incidence in a diverse HCC risk group, cirrhotic patients recently cured of hepatitis C virus (HCV) infection.

**Methods:**

In this observational cohort study, all participants (*n* = 357) had advanced fibrosis/cirrhosis and were cured of HCV with antiviral treatment. None had Liver Imaging Reporting and Data System (LI‐RADS) 2–5 lesions prior to HCV cure. Ultrasound, computed tomography, and/or magnetic resonance imaging were used for surveillance.

**Results:**

At a median follow‐up of 40 months [interquartile range (IQR) = 28–48], the median percentage of time up‐to‐date with surveillance was 49% (IQR) = 30%–71%. The likelihood of receiving a first surveillance examination was not significantly associated with race/ethnicity, but was higher for patients with more advanced cirrhosis, for example, bilirubin [odds ratio (OR) = 3.8/mg/dL, *p* = 0.002], private insurance (OR = 3.4, *p* = 0.006), and women (OR = 2.3, *p* = 0.008). The likelihood of receiving two or three examinations was significantly lower for non‐Hispanic Blacks and Hispanics versus non‐Hispanic Whites (OR = 0.39, and OR = 0.40, respectively, *p* < 0.005 for both) and for patients with higher platelet counts (OR = 0.99/10,000 cells/µl, *p* = 0.01), but higher for patients with private insurance (OR = 2.8, *p* < 0.001). Incident HCC was associated with higher bilirubin (OR = 1.7, *p* = 0.02) and lower lymphocyte counts (OR = 0.16, *p* = 0.01).

**Conclusions:**

Contrary to best practices, HCC surveillance was associated with sociodemographic factors (insurance status and race/ethnicity) among patients cured of HCV. Guideline‐concordant surveillance is needed to address healthcare disparities.

## Graphical abstract

Many hepatocellular carcinomas (HCCs) detected in patients cured of hepatitis C virus infection were present prior to cure, but did not have the characteristic imaging features needed for HCC diagnosis. Patients with worrisome imaging findings need to be excluded from measurement of HCC monitoring rates because of their potential for bias. Among patients with no worrisome imaging findings, Black and Hispanic patients were less likely to receive serial HCC surveillance than White patients, highlighting the need to reduce barriers to HCC surveillance among members of minority populations.

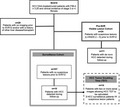

## 1 INTRODUCTION

In the United States, hepatocellular carcinoma (HCC) incidence and mortality vary by sociodemographic factors and disproportionally affect racial/ethnic minority populations. Incidence and mortality are over twofold higher among non‐Hispanic Black patients than among non‐Hispanic Whites.^1^, ^2^ The overall 5‐year survival rate of HCC is very low, with rates of <12% reported,^3^ but survival can reach 80% if HCCs are diagnosed when they are ≤2 cm in diameter.^4^

To increase early detection, the American Association for the Study of Liver Disease (AASLD) recommends lifelong twice‐annual HCC surveillance with abdominal ultrasound (US) for patients whose estimated annual HCC incidence exceeds 1%–2%,^5^, ^6^ which includes patients with cirrhosis. A recent French study showed improved survival for patients whose imaging tests were less than 7 months apart^7^ and a study from the United States had similar results,^8^ emphasizing the importance of testing at closely spaced intervals. However, only a small percentage of cirrhotic patients undergo twice‐annual imaging in the United States. In a cohort of 541 cirrhotic patients, 34% did not undergo any surveillance.^9^ In a second cohort of 904 patients, less than 2% had imaging every 6 months.^10^ Uninsured patients and Black patients are less likely to receive HCC surveillance.^10^, ^11^ Hepatitis C virus (HCV)‐infected patients have especially low rates of retention in surveillance.^12^

While the AASLD recommends US for HCC *surveillance*, computed tomography (CT) or magnetic resonance imaging (MRI) is used for *diagnosis*. The Liver Reporting and Data System (LI‐RADS) classifies observable findings based on their likelihood of representing an HCC or another liver malignancy. Because they are more sensitive than US, many providers use contrast‐enhanced MRI and/or CT for surveillance.^13^ In a head‐to‐head prospective study, US detected only 28% of HCCs, while MRI detected 86%.^14^ Despite superior sensitivity and specificity compared to US, MRI and CT are often unable to discern early HCCs.^15^, ^16^, ^17^, ^18^ Thus, serial imaging is often required to reach a diagnosis.

The recommendation for twice‐annual surveillance applies to cirrhotic patients who achieve a sustained virologic response (SVR) to HCV treatment and are cured of the infection. These patients are one of the most rapidly growing HCC risk groups. Most data indicate that HCV cure reduces HCC incidence, but details about post‐SVR risk remain uncertain.^19^, ^20^, ^21^, ^22^, ^23^, ^24^, ^25^, ^26^ In a retrospective study that did not require protocol‐specified surveillance, Black patients had a lower observed incidence of de novo post‐SVR HCC than Whites, with a hazard ratio (HR) of 0.52^21^; whereas, in a prospective study of patients with chronic HCV infection who received protocol‐specified surveillance, Black patients had a twofold higher incidence of HCC.^27^ These conflicting findings highlight the need to include information about surveillance rates in studies of HCC incidence to ensure that lower surveillance is not mistaken for lower risk.

Because the timely diagnosis of HCC requires serial abdominal imaging, but little is known about surveillance patterns in patients cured of HCV, we investigated sociodemographic and biological factors associated with surveillance in a rigorously characterized group of patients who did not have any LI‐RADS 2–5 lesions in pre‐SVR images. We used this design because recent data revealed that indeterminate lesions frequently acquire the features required for LI‐RADS 5 classification during HCV treatment.^23^ We aimed to study factors associated with initiating and continuing HCC surveillance in patients who were not receiving heightened monitoring due to worrisome pre‐existing indeterminate lesions. We also investigated HCC incidence and tumor doubling time (TDT).

## 2 PATIENTS AND METHODS

### 2.1 Study outcomes

The primary outcomes were the variables associated with HCC surveillance. Secondary and tertiary outcomes included the percentage of HCCs (LI‐RADS 5 observations) that were initially classified as CT/MRI LI‐RADS 2–4, the percentage of time “up‐to‐date‐with‐surveillance” (PTUDS),^28^, ^29^, ^30^ the annual incidence of de novo post‐SVR HCC, and HCC TDT.

### 2.2 Study design and groups

This observational study was approved by the Mount Sinai Institutional Review Board. Medical records were reviewed and patients with HCV infection and advanced fibrosis/cirrhosis (stage F3 or F4) who were treated with a direct acting antiviral (DAA)‐containing regimen and achieved an SVR between 03/01/2012 and 01/01/2018 were identified. Patients monitored for surveillance (*n* = 357) who met AASLD criteria for HCC surveillance in their providers’ clinical judgment.^5^ All had a fibrosis‐4 (FIB‐4) score ≥3.25^31^ and clinical evidence of F3 or F4 fibrosis (determined by vibration‐controlled transient elastography, liver biopsy, imaging, laboratory tests, endoscopy, and/or provider assessment based on these factors) and they did not have any LI‐RADS 2–5 observations in the last pre‐SVR imaging test. Monitoring included primary care and/or specialty practice visits, laboratory testing, and imaging. Twenty‐four patients were excluded from the surveillance group because they had LI‐RADS 2–5 lesions on pre‐SVR MRI and/or CT images; these patients were included in an analysis of TDT. An additional 29 patients were excluded from the entire study because they lacked liver imaging prior to SVR or had a history of or presented with HCC prior to DAA treatment, liver transplantation, HIV infection, or any additional liver disease.

### 2.3 HCC surveillance and tumor doubling time (TDT)

Selection of the imaging modality used for surveillance was at the discretion of the provider in the clinical setting and included any combination of US, contrast‐enhanced MRI, and CT. All images were reviewed by a single expert abdominal radiologist (SL, with 10 years of experience) using the LI‐RADS system for classification.^32^ All patients had ≥8 months of follow‐up after V_0_ (the last date images were obtained prior to SVR12). V_0_ dates ranged from 09/10/2020 to 09/07/2017. Follow‐up ended 06/01/2018. Supplementary Methods present the variables collected, LI‐RADS categories, and methods for calculating PTUDS,^28^, ^29^, ^30^ HCC incidence, and TDT.^33^

### 2.4 Statistical analysis

Logistic regressions were used to assess factors associated with participation in HCC surveillance and incident HCC. Factors with *p*‐values <0.05 were included in multivariable logistic regression (MVL) models. Factors with co‐linearity were analyzed in separate MVLs. Unpaired student's *t* tests were used to compare doubling time in months by HCV treatment stage. All analyses were performed using IBM SPSS Statistics 22.

## 3 RESULTS

### 3.1 HCC surveillance

**FIGURE 1:**
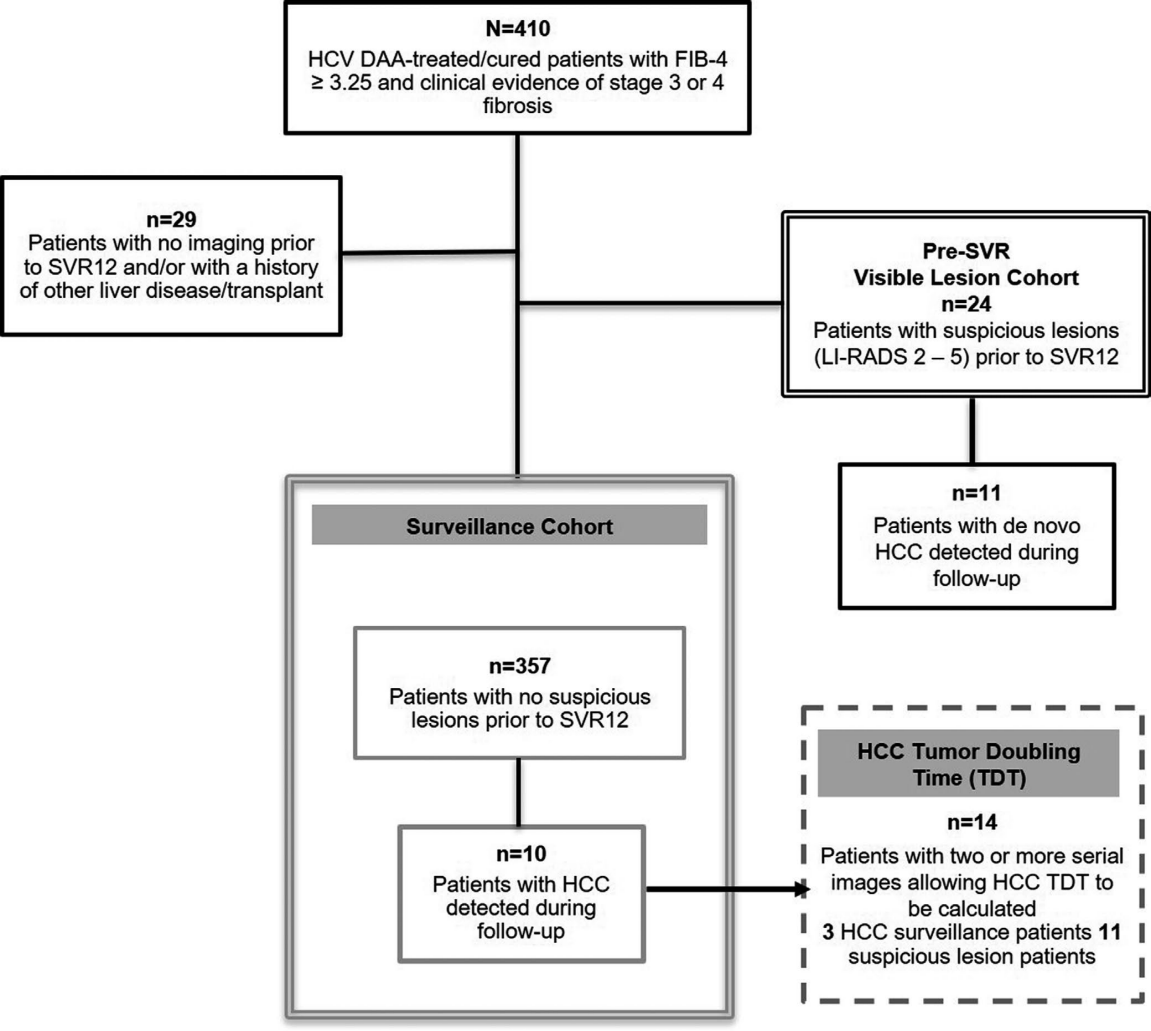
Diagram of the study groups. Records of 410 patients who were treated with regimens that contained direct acting antiviral (DAA) drugs, achieved SVR, had a FIB‐4 score ≥3.25, and whose provider recommended HCC surveillance were reviewed. Surveillance was monitored in 357 patients

**FIGURE 2:**
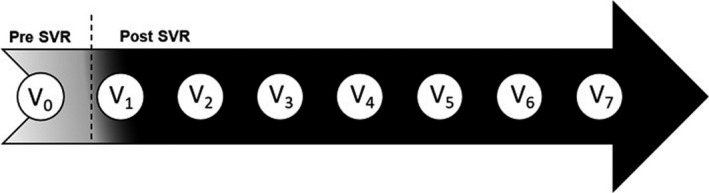
Design of surveillance monitoring, denoting the pre‐SVR (V_0_) and the post‐SVR (V_1_–V_7_) periods. V_0_, was the last HCC imaging test performed prior to SVR

**FIGURE 3:**
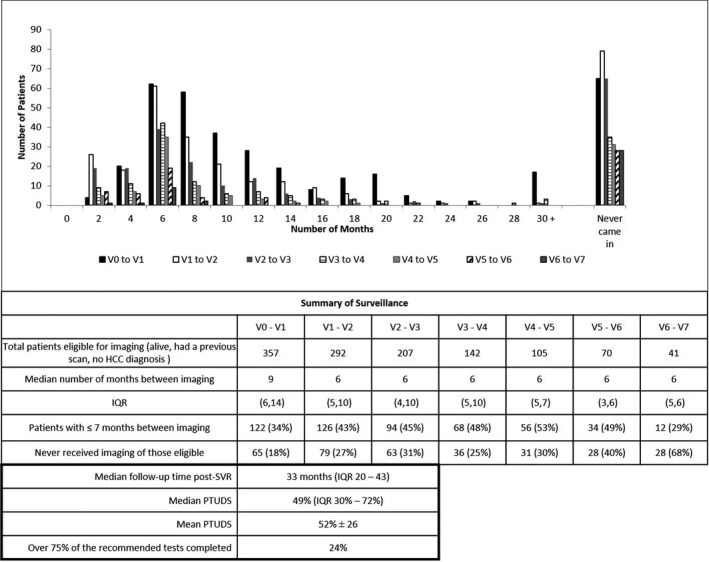
Timing of serial HCC surveillance testing. The top graph shows the time (in months) tests were performed and gives the number of patients completing each test. Serial tests are distinguished from each other by the shading of the bar, indicated in the key. V_0_–V_1_ is the interval between the last imaging test performed prior to SVR and the first post‐SVR imaging test (black bars). Summary data are reported below. PTUDS, percent time up‐to‐date surveillance; IQR, interquartile range

The cohort of 357 post‐SVR patients was 22% non‐Hispanic Black and 28% Hispanic; the majority was male; and the mean FIB‐4 score was 7.1 ± 4.6 (Figure 1; Table 1). Surveillance was monitored from V_0_ (the last date images were obtained prior to SVR) onward. The median follow‐up was 40 months [interquartile range (IQR) = 28–48] (Figures 2 and 3). Eighty‐two percent of the cohort (292 patients) had ≥1 post‐SVR imaging test. Initial post‐SVR imaging was US in 38%, MRI in 37%, and CT in 23% (Table 1). The median interval between V_0_ and V_1_ was 9 months (IQR = 6–14), which was slightly longer than subsequent intervals (Figure 3). The median PTUDS was 49% (IQR = 30–72). Only 24% of the cohort received ≥75% of the recommended tests (Figure 3).

**TABLE 1:** Characteristics of the surveillance cohort (*n* = 357)

Age, years, mean ± SD	62 ± 8.9
Male, n (%)	216 (61%)
Race/ethnicity	
White, Non‐Hispanic	121 (33.9%)
Black, Non‐Hispanic	80 (22.4%)
Other, Hispanic	101 (28.3%)
Other, Non‐Hispanic	55 (15.4%)
Mean FIB−4 score	7.1 ± 4.58
Platelets (150–450 × 10^3^ platelets/μl)^a^	10.7 ± 4.04
Albumin (3.5–5.5 g/dl)^a^	3.73 ± 0.56
Total bilirubin (0.1–1.2 mg/dl)^a^	1.05 ± 0.83
BMI (18.5–24.9 kg/m^2^)^a^	28.39 ± 5.04
Seen by liver specialist at baseline	292 (82%)
Initial post‐SVR visit imaging type	
CT	69 (23%)
MRI	110 (37%)
US	114 (38%)

Abbreviations: BMI, body mass index; CT, computed tomography; MRI, magnetic resonance imaging; US, ultrasound.

^a^ Normal range.

Factors associated with initiating and continuing surveillance post‐SVR are shown in Tables 2, 3, 4. Three MVL models were needed to identify factors independently associated with receiving at least one post‐SVR test due to co‐linearity among bilirubin, platelets, and FIB‐4 scores. Initiating surveillance was independently associated with female sex, higher bilirubin, lower platelets, and higher FIB‐4 scores. Private insurance was a significant independent factor in models that adjusted for platelets and FIB‐4 scores (Table 3). In a separate MVL of baseline factors associated with having private insurance, odds ratios (ORs) were lower for Black patients [OR = 0.18, 95% confidence interval (CI): 0.1–0.4, *p* < 0.001] and other/Hispanic patients (OR = 0.14, CI: 0.1–0.3, *p* < 0.001) than for Whites.

**TABLE 2:** Binary logistic regression analysis of factors related to receiving at least one post‐SVR surveillance test

	Mean (SD)/n (%)		Logistic regression		
	Yes, attended *n* = 292	No, did not attend *n* = 65	Odds ratio	95% confidence interval	*p*
Gender (females)	125 (42.8%)	16 (24.6%)	2.29	(1.25, 4.22)	0.008
Age (years)	62 (8.8)	62 (9.1)	1.00		0.76
Diabetes (present)	70 (26.2%)	10 (20.4%)	1.42		0.36
BMI (18.5–24.9 kg/m^2^)	28.4 (5.0)	27.7 (5.5)	1.03		0.50
Insurance (private)	75 (25.7%)	6 (9.2%)	3.40	(1.41, 8.19)	0.006
Race					
White, Non‐Hispanic (ref)	106 (55.2%)	15 (23.1%)			0.151
Black, Non‐Hispanic	63 (21.6%)	17 (26.2%)	0.52		0.10
Other, Hispanic	77 (26.4%)	24 (36.9%)	0.45		0.03
Other, Non‐Hispanic	46 (15.8%)	9 (13.8%)	0.72		0.50
Post‐SVR labs					
Total bilirubin (0.1–1.2 mg/dl)^a^	0.95 (0.83)	0.58 (0.53)	3.78	(1.63, 8.76)	0.002
Platelets (150–450 × 10^3^ platelets/μL)^a^	11.5 (4.9)	13.8 (4.8)	0.99	(0.98, 0.997)	0.003
AST (10–40 U/L)^a^	34.5 (24.2)	32.7 (15.9)	1.01		0.61
ALT (7–56 U/L)^a^	28.1 (25.8)	24.4 (18.0)	1.01		0.33
Albumin (3.5–5.5 g/dl)^a^	3.9 (0.56)	3.8 (0.53)	1.38		0.23
AFP (0.0–9.0 ng/ml)^a^	5.81 (10.6)	4.57 (2.54)	1.05		0.50
Creatinine (0.70–1.30 mg/dl)^a^	1.4 (6.81)	1.0 (0.38)	1.03		0.78
FIB‐4	5.2 (8.76)	3.5 (1.93)	1.18	(1.02, 1.38)	0.03

Abbreviations: AFP, alpha fetoprotein; ALT, alanine aminotransferase; AST, aspartate aminotransferase; BMI, body mass index; FIB‐4, fibrosis‐4 index for liver fibrosis.

^a^ Normal range.

**TABLE 3:** Multivariable logistic regression analysis of factors related to receiving at least one post‐SVR surveillance test

	Model 1			Model 2			Model 3		
	OR	95% CI	*p*	OR	95% CI	*p*	OR	95% CI	*p*
Gender (females)	2.8	(1.36, 5.60)	0.004	2.6	(1.3, 5.4)	0.008	2.4	(1.2, 4.8)	0.02
Insurance (private)	2.5		0.05	2.55	(1.03, 6.35)	0.04	2.7	(1.09, 6.73)	0.03
Post‐SVR labs									
Total bilirubin (0.1–1.2 mg/dl)^a^	4.4	(1.83, 10.72)	0.001						
Platelets (150–450 × 10^3^ platelets/μl)^a^				0.99	(0.98, 0.997)	0.004			
FIB‐4							1.19	(1.02, 1.39)	0.02

^a^ Normal range.

**TABLE 4:** Multivariable logistic regression analysis of factors related to completing two or three post‐SVR surveillance tests

	Mean (SD)/n (%)		Logistic regression^b^			Multivariable		
	Yes, attended *n* = 145	No, did not attend *n* = 147	OR	95% CI	*p*	OR	95% CI	*p*
Gender (females)	89 (61%)	81 (54%)	0.75		0.23			
Age	61 (8.6)	63 (8.9)	1.02		0.10			
Diabetes (present)	34 (24.1%)	36 (28.6%)	0.78		0.36			
BMI (18.5–24.9 kg/m^2^)	28.9 (4.8)	27.6 (5.1)	1.05		0.08			
Insurance (private)	51 (35.2%)	24 (16.3%)	2.78	(1.60, 4.84)	<0.001	2.14	(1.18, 3.90)	0.01
Race								
White, Non‐Hispanic (ref)	65 (44.5%)	42 (28.0%)			0.004			0.15
Black, Non‐Hispanic	25 (17.1%)	39 (26.0%)	0.39	(0.20, 0.74)	0.004	0.55		0.09
Other, Hispanic	30 (20.5%)	48 (32.0%)	0.40	(0.22, 0.74)	0.003	0.51		0.03
Other, Non‐Hispanic	26 (17.8%)	21 (14.0%)	0.82		0.58	0.78		0.51
Post‐SVR labs								
Total bilirubin (0.1–1.2 mg/dl)^a^	1.02 (0.76)	0.89 (0.89)	1.21		0.21			
Platelets (150–450 × 10^3^ platelets/μl)^a^	10.7 (4.8)	12.3 (4.8)	0.99	(0.988, 0.998)	0.01	0.99	(0.989, 1)	0.04
AST (10–40 U/L)^a^	34.5 (14.17)	34.6 (30.94)	1.0		0.97			
ALT (7–56 U/L)^a^	26.7 (14.48)	29.6 (33.2)	0.99		0.37			
Albumin (3.5–5.5 g/dl)^a^	3.9 (0.54)	3.9 (0.58)	0.95		0.80			
AFP (0.0–9.0 ng/ml)^a^	5.3 (2.9)	5.6 (10.88)	1.01		0.76			
Creatinine (0.70–1.30 mg/dl)^a^	0.97 (0.34)	1.77 (9.51)	0.95		0.71			
FIB‐4	4.96 (3.12)	4.74 (8.67)	0.99		0.64			

Abbreviations: AFP, alpha fetoprotein; ALT, alanine aminotransferase; AST, aspartate aminotransferase; BMI, body mass index; FIB‐4, fibrosis‐4 index for liver fibrosis.

^a^ Normal range.

^b^ Patients who completed two or three post‐SVR imaging tests were compared to patients who were eligible for these tests but did not complete them.

As determined by bivariate logistic regression, the ORs for receiving a second or third imaging test were also higher for patients with private insurance (OR = 2.8, 95% CI: 1.60–4.84) and lower for patients with higher platelets (OR = 0.99 per 10^3^/μl, CI: 0.988–0.998), and for Black (OR = 0.39, CI: 0.20–0.74) and Hispanic patients (OR = 0.40, CI: 0.22–0.74) compared to non‐Hispanic White patients (Table 4). In an MVL model, only private insurance (*p* = 0.01) and platelet counts (*p* = 0.04) were significantly associated with receiving a second or third test (Table 4).

### 3.2 HCC arising *de novo* post‐SVR12

**FIGURE 4:**
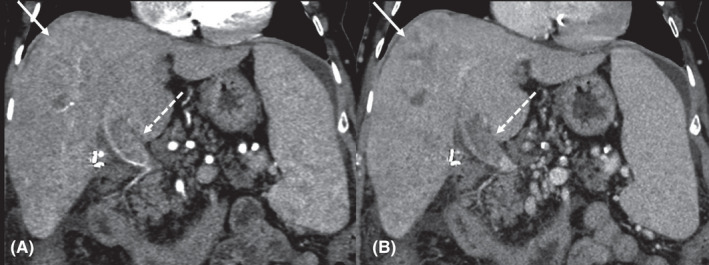
Image of a large HCC. Images from a contrast‐enhanced CT scan showing an HCC in a 66‐year‐old female cured of HCV using DAAs (Case 10). On CT in the coronal plane, an 11.4 cm ill‐defined HCC is present in the right lobe (arrows) on arterial (A) and portal venous phase (B). Enhancing portal vein tumor thrombus is also noted (dashed arrows). These findings were consistent with infiltrative HCC (LI‐RADS tumor‐in‐vein). Sequelae of portal hypertension, including splenomegaly and small volume abdominal ascites are also present; white dots are from cholecystectomy clips

**FIGURE 5:**
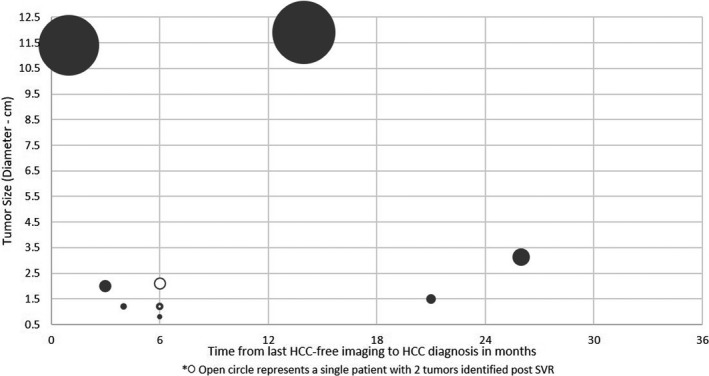
Tumor size plotted with time between the last scan that did not contain a LI‐RADS 5 lesion and the diagnostic scan. Tumor diameter (scaled to size) at HCC diagnosis related to the time between the previous (HCC free) imaging and HCC diagnosis, in months. The asterisk identifies lesions in a patient who had two de novo LI‐RADS 5 lesions post‐SVR

Ten (2.8%) of the 357 patients developed a total of 11 HCCs (Table S1A‐B). The HCC incidence rate was 1.6 per 100 person‐years. After confirming no significant difference by disease severity (FIB‐4), a survival analysis (Kaplan–Meier—log‐rank test) assessing differences in HCC incidence determined that those with PTUDS ≥75% had significantly higher likelihood of developing HCC (x^2^ = 25.32, *p* < 0.001). Blacks were more likely to develop HCC (OR = 1.53, *p* = 0.61), but the difference was not statistically significant. Eight HCCs (73%) detected in scans obtained within 6 months of the previous scan were ≤2.5 cm at diagnosis; the others were 3.1, 11.4, and 11.9 cm. The largest was diagnosed by CT (Figure 4). The previous test was an US performed 14 months earlier. The relationship between the time between scans and tumor diameter is presented in Figure 5. Baseline and post‐SVR factors associated with de novo HCC are presented in Table S2.

### 3.3 LI‐RADS 5 lesions initially classified as LI‐RADS 3 or 4 and HCC doubling times

To determine the percentage of HCCs initially classified as CT/MRI LI‐RADS 3–4, we combined data on the 11 HCCs that developed in 10 patients in the surveillance cohort with data on 14 HCCs that developed in 11 of the 24 patients who had indeterminate/suspicious lesions in V_0_ images and thus were excluded from the surveillance cohort (see Figure 1). Eighteen of the 25 HCCs (72%) were initially classified as LI‐RADS 3 or 4.

**FIGURE 6:**
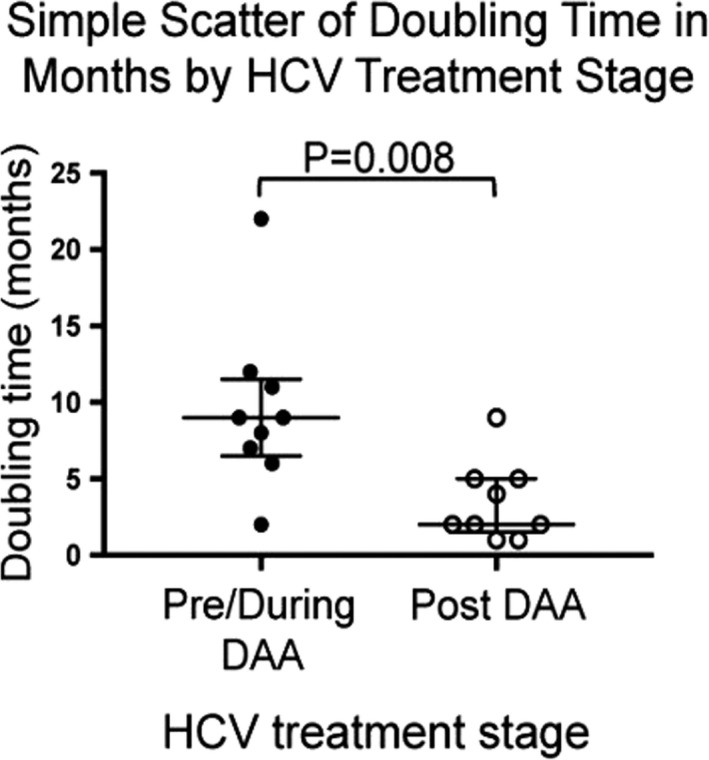
Scatterplot of HCC doubling times in tumors detected before and after the end of treatment. Using serial images, the doubling time of LI‐RADS 3–4 lesions that were later classified as LI‐RADS 5 observations before or during DAA treatment were compared to those detected after the end of treatment. Data were analyzed using an unpaired *t* test

TDT was calculated on 18 HCCs present in two or more serial images. Three arose in patients in the surveillance cohort and 11 in patients who had LI‐RADS 2–5 observations in pre‐SVR (V_0_) images (Figure 1). The average TDT of HCC lesions detected prior to or during DAA treatment was 9.5 ± 5.5 months, significantly longer than the TDT of HCCs detected post‐SVR (3.4 ± 2.6 months; *p* < 0.008; Figure 6).

## 4 DISCUSSION

HCC surveillance has the potential to save lives but is a demanding process that involves serial abdominal imaging at 6‐month intervals for life. Ideally, the patients who are most likely to benefit are the ones most likely to undergo surveillance. We found that HCC surveillance was related to both a patient's liver disease status, as indicated by low platelet counts, and to sociodemographic factors, such as insurance coverage. The latter reveals a disconnection between HCC risk and HCC surveillance that is especially relevant to US populations. Patients with private insurance and White patients were more likely to receive two or three surveillance tests than Black patients, even though Black patients are more likely to present with advanced HCC and to die from it.^34^, ^35^, ^36^, ^37^ Unlike a prior study, which showed a greatly reduced risk of post‐SVR HCC in Black patients (HR = 0.52),^21^ we found an increased risk (OR = 1.53). This finding suggests that the HCC risk in Black patients cured of HCV may be higher than reported, although our findings were not statistically significant and warrant further investigation. Black patients present with more advanced HCC than Whites, but are younger, have better liver function, and are less likely to have cirrhosis.^34^, ^35^, ^36^, ^37^ Additional studies are needed to determine whether surveillance guidelines should be adjusted to account for the tendency of HCC to arise in African American patients with relatively well‐preserved liver function.^38^

Our findings confirmed data showing that insurance status (private vs. others), race, and socioeconomic status are associated with HCC surveillance.^9^, ^10^, ^28^, ^39^ After adjusting for gender and liver status, private insurance had ORs ranging from 2.5 to 2.7 in our study. Black and Hispanic patients were less likely to have private insurance than Whites. To our knowledge, our study is the first to examine gender in post‐SVR HCC surveillance; our findings accord with the higher participation of women reported in other settings.^28^, ^40^

Nationally, as few as 18.4% of cirrhotic patients receive surveillance in the United States,^41^ with variable rates reported for HCV‐infected patients.^12^, ^30^ Over 80% of patients in our surveillance cohort received at least one post‐SVR imaging test. Our PTUDS was 49%; however, only 24% received ≥75% of the recommended tests. Our findings underscore the need to identify barriers that keep patients, especially men and members of racial and ethnic minority populations, from undergoing surveillance, as noted before.^42^ Simple interventions, such as sending patients reminders, can improve testing.^43^

Our study reveals the likely benefits of HCC surveillance in cirrhotic patients cured of HCV. The annual incidence was about 1.6%, consistent with other studies,^3^, ^26^, ^44^ and above the AASLD threshold for twice‐annual imaging. Most HCC was detected at an early and potentially curable stage. Patients who developed de novo HCC had higher bilirubin and lower lymphocytes both at baseline and after cure; these variables might help predict post‐SVR HCC risk.

We confirmed the findings of Marino et al.^23^ and demonstrated that a high percentage of indeterminate lesions are later re‐classified as LI‐RADS 5. Eleven of 24 patients (46%) with suspicious lesions prior to HCV cure subsequently received a diagnosis of HCC compared to only 10 of 357 patients (3%) who did not have suspicious lesions. This difference underscores the importance of reviewing pre‐SVR scans (and excluding patients with existing lesions) when attempting to determine the impact of HCV cure on HCC incidence. Our observed HCC incidence rate would have been about twofold higher had we included patients with pre‐existing (but undiagnosed) HCCs. Nearly three‐quarters of HCCs were visible before they met diagnostic LI‐RADS 5 criteria, somewhat higher than previously reported.^17^ Many HCCs could be detected by imaging more than a year before they acquired the features needed for a definitive diagnosis, delaying treatment, and allowing time to spread.

The doubling time of lesions first observed post‐SVR was shorter than the doubling time of lesions detected before or during antiviral treatment. This finding contrasts with data from Toyoda et al., who found HCV cure did not impact HCC growth.^45^ However, 72.1% of their patients did not have cirrhosis, all patients underwent gadoxetic‐enhanced MRI, and only lesions which would constitute LI‐RADS 4 lesions (had LI‐RADS criteria been applied) were assessed.

In this study, the number of HCCs included in the calculation of TDT was small and imaging modalities were not uniform, which can introduce bias. Large multicenter studies are needed to rigorously test the hypothesis that HCCs grow more rapidly in the immediate aftermath of HCV eradication.

The strengths of our study include our monitoring of surveillance in patients in the clinical setting whose pre‐SVR liver images were reviewed rigorously to exclude patients with LI‐RAD 2–5 observations, which was not performed in most studies.^46^, ^47^, ^48^ Additional strengths included the extended follow‐up time and the use of PTUDS for reporting surveillance.^28^, ^29^, ^30^

The limitations include the single‐site design, which might reduce generalizability; however, the cohort was racially and ethnically diverse. Our estimate of HCC incidence is inexact because imaging can miss early HCCs and because some HCCs might have gone undetected as a result of incomplete surveillance. Our ~50% PTUDS could be an underestimate if patients had imaging at other institutions. Our relatively small number of cases limits our ability to identify factors independently associated with HCC development.

Conclusions: A high percentage of HCCs diagnosed in patients recently cured of HCC were pre‐existing, but undiagnosed, HCCs. Future studies of post‐SVR HCC incidence should exclude patients with pre‐existing lesions and should adjust for differences in surveillance testing among racial, ethnic, and socioeconomic groups. This will allow lower rates of HCC incidence to be distinguished from lower rates of HCC detection. Interventions are needed to increase surveillance in men, members of minority populations, and patients without private insurance.

## CONFLICT OF INTEREST

The authors declare no conflict of interest.

## DISCLOSURE

Mount Sinai receives research from Gilead Sciences to support Dr. Branch's research. There are no other relevant financial or personal disclosures to report on behalf of the authors. All authors contributed to the manuscript.

## AUTHOR CONTRIBUTION

All authors contributed to the drafting and publication of this manuscript.

## Supporting information

Table S1‐2

Supplementary Material

## Data Availability

Data available upon request.
