# Supplementary material for: Hepatocellular carcinoma surveillance, incidence, and tumor doubling times in patients cured of hepatitis C
Source: Cancer Med. 2022 Mar 9;11(9):1995–2005. doi: 10.1002/cam4.4508 (PMC9089228; doi:10.1002/cam4.4508)
Supplement: Supplementary file 1 — Table S1‐2 [file CAM4-11-1995-s001.docx]

**Supplemental Table 1A. Characteristics of ten patients who developed de novo HCC after SVR.**

| **Case**  **#** | **FIB-4*** | **Total Bilirubin**  (0.1 to 1.2 mg/dL) ***** | **Platelets**  (150 to 450 × 10^3^/μL)***** | **Months from last HCC-free scan to Dx** | **Number of post-SVR imaging tests at Dx** | **Age at Dx (yr)** | **Number of HCCs** | **Size of HCC (cm)** | **HCC treatment** |
| --- | --- | --- | --- | --- | --- | --- | --- | --- | --- |
| 1** | 4.8 | 3.7 | 16.0 | 1 | 5 | 56 | 1 | 2.1 | TACE/RFA; awaiting transplant |
| 2 | 13.4 | 3 | 7.1 | 6 | 1 | 61 | 1 | 1.2 | TACE; transplanted |
| 3** | 23.7 | 2.4 | 5.0 | 6 | 4 | 64 | 2 | 1.2, 2.1 | TACE; transplanted |
| 4 | 6.1 | 0.5 | 10.3 | 6 | 2 | 72 | 1 | 1.5 | Y90 |
| 5 | 6.3 | 0.6 | 9.1 | 21 | 2 | 60 | 1 | 1.2 | Resection |
| 6 | 13.4 | 2.1 | 6.2 | 4 | 1 | 71 | 1 | 3.1 | TACE, RFA |
| 7 | 5.6 | 1.1 | 6.6 | 26 | 1 | 66 | 1 | 11.9 | Awaiting transplant |
| 8** | 27.5 | ND | 2.8 | 14 | 3 | 62 | 1 | 2 | Died while awaiting transplant |
| 9 | 10.8 | 1.5 | 8.5 | 3 | 4 | 62 | 1 | 0.8 | TACE, RFA |
| 10 | 7.3 | 1.2 | 9.4 | 6 | 4 | 66 | 1 | 11.4 | Y90; under evaluation |
| **Supplemental Table 1B: Summary data** | | | | | * Pretreatment lab values and normal ranges if appropriate  ** Patients with two or more serial imaging exams where HCC TDT could be calculated  AST = aspartate transaminase; ALT = alanine transaminase; Dx = diagnosis of HCC; TACE = transcatheter arterial chemoembolization; RFA = radiofrequency ablation; Y90 = radioembolization; ND = no data | | | | |
| Male | | | 6 (60%) | |  |  |  |  |  |
| White | | | 3 (30%) | |  |  |  |  |  |
| Black | | | 3 (30%) | |  |  |  |  |  |
| Mean FIB-4 Score at baseline | | | 11.89 ± 7.92 | |  |  |  |  |  |
| HCV Genotype 1a/1b | | | 10 (100%) | |  |  |  |  |  |
| Median time to HCC diagnosis post V_0_ | | | 22.5 months  (IQR 14.3–32.8) | |  |  |  |  |  |
| Mean HCC diameter | | | 3.5 ± 4.1 cm | |  |  |  |  |  |
| HCC ≤ 2.5 cm | | | 8/11 (73%) | |  | | | | |

**Supplemental Table 2. Logistic regression of baseline and post-SVR factors associated with the development of *de novo* HCC during surveillance.**

|  |  |  | | **Logistic Regression*** | | |
| --- | --- | --- | --- | --- | --- | --- |
|  | **Total**  **n=357** | **Mean (SD) / n (%)** | | **OR** | **95% CI** | **P-Value** |
|  |  | **No HCC, n=347** | **HCC, n=10** |  |  |  |
| **Gender** (male) | 216 (61%) | 210 (61%) | 6 (60%) | 1.02 |  | 0.97 |
| **Age** | 62 (8.86) | 62 (8.95) | 63 (5.10) | 1.01 |  | 0.82 |
| **Diabetes** (present) | 80 (22.2) | 78 (25%) | 2 (20%) | 0.82 |  | 0.81 |
| **BMI** (18.5 to 24.9 kg/m^2^) * | 28.39 (5.04) | 28.4 (5.12) | 27.9 (1.63) | 0.98 |  | 0.78 |
| **Insurance** (private) | 81 (22.7%) | 76 (22%) | 5 (50%) | 5.6 |  | 0.05 |
| **Race/Ethnicity**  White, Non-Hispanic (ref)  Black, Non-Hispanic  Other, Hispanic  Other, Non-Hispanic | 121 (33.9%)  80 (22.4%)  101 (28.3%)  55 (15.4%) | 118 (34%)  77 (22.2%)  99 (28.5%)  53 (15.3%) | 3 (30%)  3 (30%)  2 (20%)  2 (20%) | 1.53  0.79  1.48 |  | 0.88  0.61  0.80  0.67 |
| **Pre-Treatment Labs** |  |  |  |  |  |  |
| Total Bilirubin (0.1 to 1.2 mg/dL) | 1.05 (0.83) | 1.02 (0.81) | 1.8 (1.01) | 1.66 | (1.07, 2.58) | 0.02 |
| Platelets (150 to 450 × 10^3^ platelets/μL) | 10.7 (4.04) | 10.8 (4.04) | 8.1 (3.6) | 0.98 |  | 0.05 |
| FIB-4* | 7.1 (4.58) | 6.9 (4.38) | 11.89 (7.92) | 1.12 | (1.04, 1.24) | 0.004 |
| AST (10 to 40 U/L) | 98.6 (66.86) | 98.4 (66.95) | 114.9 (64.60) | 1.00 |  | 0.44 |
| ALT (7 to 56 U/L) | 93.1 (79.05) | 93.3 (79.60) | 87.3 (59.60) | 1.00 |  | 0.81 |
| Albumin (3.5 to 5.5 g/dL) | 3.73 (0.56) | 3.7 (0.56) | 3.4 (0.56) | 0.44 |  | 0.12 |
| AFP (0.0 to 9.0 ng/mL) | 25.6 (49.34) | 25.9 (49.89) | 16.2 (19.22) | 0.99 |  | 0.62 |
| Creatinine (0.70 to 1.30 mg/dL) | 0.99 (0.48) | 0.99 (0.49) | 0.98 (0.24) | 0.95 |  | 0.96 |
| Neutrophil (1.9 to 8.0 x10^3^/uL) | 2.9 (1.36) | 2.9 (1.35) | 2.85 (1.56) | 0.97 |  | 0.91 |
| Lymphocyte (1.0 to 4.5 x10^3^/uL) | 1.66 (1.08) | 1.7 (1.08) | 0.9 (0.48) | 0.16 | (0.04, 0.61) | 0.01 |
| Neutrophil/Lymphocyte Ratio | 2.19 (1.47) | 2.2 (1.48) | 3.1 (1.07) | 1.30 |  | 0.07 |
| **Post-SVR Labs** |  |  |  |  |  |  |
| Total Bilirubin (0.1 to 1.2 mg/dL) | 0.898 (0.81) | 0.9 (0.80) | 1.6 (0.80) | 1.67 | (1.08, 2.58) | 0.02 |
| Platelets (150 to 450 × 10^3^ platelets/μL) | 11.8 (4.9) | 11.9 (4.9) | 7.7 (3.33) | 0.98 | (0.96, 0.99) | 0.01 |
| FIB-4 | 4.96 (8.17) | 4.9 (8.25) | 7.6 (4.51) | 1.02 |  | 0.35 |
| AST (10 to 40 U/L) | 34.3 (23.24) | 33.93 (23.14) | 45.1 (25.08) | 1.01 |  | 0.20 |
| ALT (7 to 56 U/L) | 27.55 (24.9) | 27.3 (24.87) | 35.9 (24.7) | 1.01 |  | 0.31 |
| Albumin (3.5 to 5.5 g/dL) | 3.9 (0.56) | 3.9 (0.55) | 3.6 (0.73) | 0.46 |  | 0.11 |
| AFP (0.0 to 9.0 ng/mL) | 5.69 (10.06) | 5.6 (10.17) | 8.2 (5.54) | 1.01 |  | 0.46 |
| Creatinine (0.70 to 1.30 mg/dL) | 1.3 (6.29) | 1.3 (6.38) | 1.0 (0.23) | 0.97 |  | 0.91 |
| Neutrophil (1.9 to 8.0 x10^3^/uL) | 3.3 (1.57) | 3.3 (1.57) | 2.6 (1.26) | 0.70 |  | 0.25 |
| Lymphocyte (1.0 to 4.5 x10^3^/uL) | 1.68 (0.89) | 1.7 (0.89) | 0.9 (0.40) | 0.16 | (0.038, 0.68) | 0.01 |
| Neutrophil/Lymphocyte Ratio | 2.4 (1.78) | 2.4 (1.79) | 3.0 (1.20) | 1.12 |  | 0.41 |
| * Post-SVR patients who did not develop *de novo* HCC were compared to patients who did develop *de novo* HCC during follow-up. | | | | | | |
