# Supplementary material for: Hepatocellular carcinoma surveillance, incidence, and tumor doubling times in patients cured of hepatitis C
Source: Cancer Med. 2022 Mar 9;11(9):1995–2005. doi: 10.1002/cam4.4508 (PMC9089228; doi:10.1002/cam4.4508)
Supplement: Supplementary file 2 — Supplementary Material [file CAM4-11-1995-s002.docx]

Supplemental text.

Supplemental Methods

**LI-RADS categories.** US LI-RADS: 1 (negative), 2 (subthreshold), and 3 (positive); US LI-RADS 3 observations require contrast-enhanced CT or MRI for diagnosis. CT/MRI LI-RADS categories are: 1 (definitely benign), 2 (probably benign), 3 (indeterminate/lacks features needed to categorize as likely benign or likely malignant), 4 (probably HCC), and 5 (HCC) and tumor-in-vein (TIV). LIRADS-TIV was grouped with LI-RADS 5 for analysis.

**Variables analyzed**

Demographic variables included age, race/ethnicity, insurance, and sex. Clinical variables included DAA treatment regimen; comorbidities; laboratory test results immediately prior to HCV treatment and post SVR [platelets, bilirubin, albumin, aspartate transaminase (AST), alanine transaminase (ALT), alpha fetoprotein (AFP), and creatinine]; type of provider managing post-SVR care; and imaging data.

**Calculation of the percent of time up to date with surveillance (PTUDS).** A patient was considered 100% “up-to-date” for the first 6 months after each imaging test, starting at V_0_. If the interval between tests was more than 6 months, the PTUDS decreased in proportion to the time until the next test. The clock restarted at each imaging visit, even if the visits were less than 6-months apart. For example, a patient followed from 1/1/2012 to 12/31/2012, who had imaging on January 1 and August 1 would have a PTUDS of 92% (11/12 months).

**Calculation of HCC tumor doubling time (TDT)**

TDT was calculated on HCCs present in two or more serial images, as follows: TDT=(T − T_0_) × log2/logV – logV_0_, where “T” is the date for scan 2, “T_0_” is the date for scan 1, and “V_0_” is the tumor volume. If more than two serial images were available, the first and last were used. HCCs in the same patient treated as independent lesions.

**Calculation of HCC incidence.** The number of patients who developed HCC divided by the total amount of time each individual was followed and at risk for HCC. Each patient’s disease-free time was calculated as the time from study entry until HCC diagnosis, loss to follow-up occurred, or the study end date (06/01/2018). To take into consideration variability in length of follow-up and HCC surveillance, cumulative incidence was assessed by survival analysis (Kaplan-Meier – log rank test).
